# Evaluation of elderly patients with bacteremia in the cardiology intensive care unit

**DOI:** 10.1097/MD.0000000000040863

**Published:** 2024-12-20

**Authors:** Sibel Doğan Kaya, Yeşim Uygun Kizmaz, Fatih Yiğit

**Affiliations:** a Diseases and Clinical Microbiology Department, Kosuyolu High Specialization Training and Research Hospital, Istanbul, Turkey; b Heart Surgery Department, Kosuyolu High Specialization Training and Research Hospital, Istanbul, Turkey.

**Keywords:** blood culture, blood stream infections, elderly, intensive care unit

## Abstract

In this study, the blood culture results of patients aged >65 years who were admitted to the cardiology intensive care unit in a training and research hospital and who had positive blood cultures within the first 48 hours were evaluated. This was a retrospective, observational and nonrandomized study. Patient data at the time of the blood culture were included in the study. Sex, age, length of hospital stay, mortality, Acute Physiology Chronic Health Evaluation II score, laboratory values, and microorganisms grown in blood culture included in the study. Two hundred forty-seven patients, 43.3% of whom were female (n = 107), were included in the study. The median age of the patients was 75 (range 70–83). The mean hospital stay was 5 days (range 3–8). All patients had a median Acute Physiology Chronic Health Evaluation II score of 21 (range 19–23). The overall mortality rate 48.2% (n = 119). The results showed that 49.8% of those died and 50.2% of survivors had positive blood culture results. The most common gram-positive cocci in those died were *Staphylococcus hominis* (15.6%), *Staphylococcus epidermis* (14.8%), *Enterococcus faecium* (9.6%). The most common gram-negative cocci were *Escherichia coli* (9.6%), *Klebsiella pneumoniae* (9.6%), *Acinetobacter baummanii* (1.6%). With an increase in the elderly population, infection management in elderly patients hospitalized in cardiology intensive care units has become increasingly critical.

## 1. Introduction

As people age, they are more likely to experience health issues and experience difficulty adjusting to their surroundings. The elderly population has proliferated in developed countries, with a projected 140% increase in individuals aged 65 and over by 2030. The fastest-growing group is expected to be those aged ≥85 years.^[1]^ As per the population projections, the elderly population rate is set at 12.9% in 2030, 16.3% in 2040, 22.6% in 2060, and 25.6% in 2080.^[2]^ The increasing number of elderly patients admitted to the cardiac intensive care units due to demographic changes underscores the need for preparedness to manage a patient population that is more prone to cardiovascular disease (CVD).^[3]^

It is a stark reality that two-thirds of all patients with CVD are over 60 years old, and more than 85% of patients over 85 years old live with some form of CVD. The high prevalence of CVD in older patients underscores the urgent need to address this issue in geriatric care.^[4]^

According to population projections, the elderly population is expected to increase. Although CVD is a leading cause of illness and death in adults of all ages, older patients are at a higher risk of adverse outcomes. These include higher mortality rates, increased rehospitalizations, reduced quality of life, and declining physical function. Older adults with acute CVD are often transferred to cardiac intensive care units to receive the best possible care.^[5]^

Multiple factors may contribute to the increased infection rate in elderly patients, including comorbidities, exposure to devices and procedures, institutionalization, immunosuppression, malnutrition, and poor performance status. Elderly patients are at a higher risk of infection because of a more pronounced impairment of their defenses against disease and a greater risk of multiple comorbidities, as well as the rapid spread of viral infections and multidrug-resistant organisms.^[6]^

Bloodstream infection (BSI) is defined as positive blood cultures in a patient with signs of systemic disease and may be secondary or primary to a source. BSIs are among the most common infections in intensive care units. In an international study on the prevalence and outcomes of infections in intensive care units, BSIs were observed in 15% of infected patients and were the third most common infection.^[7]^

While community-acquired BSIs in immunocompetent adults usually involve drug-susceptible bacteria, healthcare-associated BSIs are often caused by multidrug-resistant strains.

This study aimed to evaluate elderly patients with bacteremia in the cardiology intensive care unit and examine the potential impact of the data obtained from this evaluation on the treatment and care of the patients.

## 2. Methods

This was a retrospective, observational, nonrandomized study conducted between January 1, 2019, and December 31, 2023. Blood culture results of patients admitted to the cardiology intensive care unit who were 65 years and older and had growth in blood culture within the first 48 hours. Patients under 65 years of age and those hospitalized for more than 48 hours were excluded from the study. In this study, if the same pathogen was isolated in at least 2 blood culture bottles simultaneously collected as a set for each patient to isolate coagulase-negative staphylococci, this microorganism was considered the causative agent. If growth occurred in only 1 out of 2 blood cultures, clinical correlation was checked, and if it was isolated from different infection sites, it was considered as the causative agent. The isolation of a single gram-negative pathogen was considered significant and was identified as the causative agent. If a species of normal skin flora was isolated from one of the blood cultures taken at the same time, it was considered contamination. If coagulase-negative staphylococci was isolated in a single blood culture from a patient and was not compatible with fever >38 °C, chills, or hypotension, this result was also considered as contamination.^[8]^

When the same agent grew in multiple blood cultures from the same patient, only the first isolated agent was evaluated. Patient data at the time of the blood culture were included in the study. Sex, age, length of hospital stay, mortality, Acute Physiology Chronic Health Evaluation II (APACHE II) score, laboratory values, C-reactive protein (CRP), glomerular filtration rate, white blood cell (WBC), platelet, mean platelet volume, procalcitonin (PCT), platelet distribution width (PDW), urea, creatinine, albumin, and microorganisms grown in blood culture (during hospitalization and within the first 48 hours after hospitalization) were included in the study. They were evaluated in the Microbiology Laboratory using the VITEK 2 (bioMerieux, France) blood culture system, and growth samples were examined using gram staining.

Ethical approval was obtained from the Kartal Kosuyolu Heart Education and Research Hospital Ethics Committee before the study (date: 24/10/2023, Decision No. 2023/16/721). This study was conducted in accordance with the principles outlined in the Declaration of Helsinki.

## 3. Statistical analysis

The statistical analysis for this study was performed using IBM SPSS Statistics software v27 (NY). Categorical variables are expressed as frequencies and percentages, whereas numerical variables are presented as medians and interquartile range (25th–75th percentiles). Kolmogorov–Smirnov and Shapiro–Wilk tests were used to assess normality. Pearson chi-square and Fisher exact tests were employed to evaluate the impact of categorical variables among independent groups. The Mann–Whitney U test was used to compare the medians of 2 independent groups that did not conform to a normal distribution. Logistic regression analysis was conducted to identify independent predictive factors for mortality. Variables with a *P*-value < .2 in the univariate analysis were subsequently included in the multivariate analysis. Statistical significance was set at *P* < .05.

## 4. Results

This study included 247 patients, of whom 43.3% (n = 107) were female (Table 1). The median age of the patients was 75 years (range 70–83). The median duration of hospital stay was 5 days (range 3–8). The median APACHE II score for all patients was 21 (range 19–23). The overall mortality rate was 48.2% (n = 119).

**Table 1:** Patients characteristics.

	% (n)/median (IQR)
Age (year)	75 (70–83)
Gender (female)	43.3% (n = 107)
Hospital stay (day)	5 (3–8)
APACHE II	21 (19–23)

IQR = interquartile range.

The distributions of patient age, sex, length of hospital stay, and laboratory variables according to mortality are shown in Table 2. There were no statistically significant differences between the groups in terms of age, sex, length of hospital stay, CRP, platelet, mean platelet volume, or PCT level with respect to mortality. However, statistically significant differences were observed between the groups in terms of APACHE II, PCT, albumin, creatinine, glomerular filtration rate, WBC, and PDW values (*P* < .05). Patients who died had higher PCT, creatinine, WBC, and PDW levels. Patients who survived had higher APACHE II and albumin levels (Table 3).

**Table 2:** Distribution of sociodemographic and laboratory variables by mortality.

	Mortality		
	Alive		Exitus
	% (n)/median (IQR)		% (n)/median (IQR)
Age	76 (72–83)		74 (69–82)
Hospital stay	5 (3–8)		5 (3–8)
APACHE II	23 (21–24)		19 (18–21)
CRP	89.15 (33.985–181.33)		106 (56.5–181)
Procalcitonin	0.385 (0–1.895)		1.19 (0.1–7.69)
Albumin	29 (20–34)		25 (18–31)
Creatinine	1.45 (0.955–2.165)		1.93 (1.14–2.64)
GFR	24 (0–54)		0 (0–27)
WBC	11.605 (7.85–16.775)		13.05 (9.24–19.5)
PLT	207.5 (138–281)		197 (138–271)
MPV	10 (9.2–10.9)		9.8 (8.6–11)
PCT	0.195 (0.14–0.29)		0.19 (0.13–0.28)
PDW	13.45 (11.4–17)		16.6 (12.2–17.5)
Gender	Female	51 (47.7%)	56 (52.3%)
	Male	77 (55.0%)	63 (45.0%)

Independent predictive factors affecting mortality were investigated in Table 3. In univariate analysis, PDW (*P*: .020, OR: 1.087; CI: 1.013–1.167), WBC (*P*: .048, OR: 1.035; CI: 1.000–1.071), GFR (*P*: .000, OR: 0.981; CI: 0.971–0.991), and APACHE II (*P*: .000, OR: 0.711; CI: 0.634–0.797) were among the independent predictors affecting mortality. In multivariate analysis, WBC (*P*: .033, OR: 1.043; CI: 1.003–1.085), GFR (*P*: .012, OR: 0.985; CI: 0.973–0.997), and APACHE II score (*P*: .000, OR: 0.713; CI: 0.634–0.801) were the independent predictive factors affecting mortality.

CRP = C-reactive protein, GFR = glomerular filtration rate, MPV = mean platelet volume, PCT = procalcitonin, PDW = platelet distribution width, PLT = platelet, WBC = white blood cell.

**Table 3:** Association of laboratory and clinical variables with mortality in sepsis.

	Univariate				Multivariate			
	*P*	OR	95% CI for EXP(B)		*P*	OR	95% CI for EXP(B)	
			Lower	Upper			Lower	Upper
PDW	**0.020**	1.087	1.013	1.167	0.278	1.047	0.964	1.136
WBC	**0.048**	1.035	1.000	1.071	**0.033**	1.043	1.003	1.085
GFR	**0.000**	0.981	0.971	0.991	**0.012**	0.985	0.973	0.997
CRP	0.103	1.002	1.000	1.005	0.200	1.002	0.999	1.005
Age	0.106	0.973	0.942	1.006	0.312	0.981	0.944	1.018
APACHE II	**0.000**	0.711	0.634	0.797	**0.000**	0.713	0.634	0.801
PCT	0.477	1.266	0.661	2.428				
MPV	0.482	1.011	0.980	1.044				
PLT	0.557	0.999	0.997	1.002				
Creatinine	0.310	1.081	0.930	1.258				
Albumin	0.387	0.992	0.973	1.010				
Procalcitonin	0.243	1.009	0.994	1.025				
Gender (female)	0.253	0.745	0.450	1.234				

Independent predictive factors affecting mortality were investigated in Table 3. In univariate analysis, PDW (*P*: .020, OR: 1.087; CI: 1.013–1.167), WBC (*P*: .048, OR: 1.035; CI: 1.000–1.071), GFR (*P*: .000, OR: 0.981; CI: 0.971–0.991), and APACHE II (*P*: .000, OR: 0.711; CI: 0.634–0.797) were among the independent predictors affecting mortality. In multivariate analysis, WBC (*P*: .033, OR: 1.043; CI: 1.003–1.085), GFR (*P*: .012, OR: 0.985; CI: 0.973–0.997), and APACHE II score (*P*: .000, OR: 0.713; CI: 0.634–0.801) were the independent predictive factors affecting mortality.

CRP = C-reactive protein, GFR = glomerular filtration rate, MPV = mean platelet volume, PCT = procalcitonin, PDW = platelet distribution width, PLT = platelet, WBC = white blood cell.

The results show that blood cultures were positive in 49.8% of deceased and 50.2% of survivors. The most common gram-positive cocci species in the deceased were *Staphylococcus hominis* (*S hominis*) (15.6%), *Staphylococcus epidermidis* (*S epidermidis*) (14.8%), *Enterococcus faecium* (*E faecium*) (9.6%), and *Staphylococcus aureus* (*S aureus*) (10.9%). The most common gram-negative cocci species were *Escherichia coli* (*E coli*) (9.6%), *Klebsiella pneumoniae* (*K pneumoniae*) (9.6%), *Acinetobacter baumannii* (1.6%), and *Pseudomonas aeruginosa* (*P aeruginosa*) (1.6%). In the survivors, the most common gram-positive cocci species were *S hominis* (23.5%), *S epidermidis* (24.3%), *E faecium* (3.3%), and *S aureus* (13.4%). The most common gram-negative cocci were *E coli* (5.4%), *K pneumoniae* (5.8%), and *P aeruginosa* (2.5%) (Table 4).

**Table 4:** Causative agents of bacteremia.

Agents	Deceased (n, %)		Alive (n, %)	
*Staphylococcus hominis*	20	15.6	28	23.5
*Staphylococcus epidermidis*	19	14.8	29	24.3
*Staphylococcus aureus*	14	10.9	16	13.4
*Enterococcus faecalis*	12	9.6	4	3.3
*Escherichia coli*	12	9.6	6	5.4
*Enterococcus faecium*	12	9.6	6	5.4
*Klebsiella pneumoniae*	12	9.6	7	5.8
*Staphylococcus haemolyticus*	5	3.9	3	2.5
*Stenotrophomonas maltophilia*	5	3.9	3	2.5
*Pseudomonas aeruginosa*	2	1.5	3	2.5
*Streptococcus mitis*	1	0.7	4	3.3
*Serratia marcescens*	2	1.5	2	1.6
*Enterobacter aerogenes*	2	1.5	2	1.6
*Sfingomonas paucimobilis*	1	0.7	2	1.6
*Streptococcus pneumoniae*	0	0	3	2.5
*Enterobacter cloacae*	2	1.5	0	0
*Acinetobacter baumannii*	2	1.5	0	0
*Streptococcus agalactiae*	2	1.5	0	0

## 5. Discussion

In our country, geriatric patients with indications for intensive care are hospitalized without age restrictions. In the literature, it has been shown that BSIs are more common in elderly patients, and more than 50% of cases occur in patients aged >65 years.^[9]^ In our study, more than one-third of the patients admitted to the cardiology intensive care unit were aged 70 years and older, and 14.4% were at least 80 years old.^[10]^ Women constituted 43.3% of the population, and this rate was similar to the female rates in other intensive care units.^[11]^ In the study by Nowak et al, 44.8% of female patients were found in the cardiology intensive care unit, with a mean age of 74.8 years.^[12]^ Our study determined that the median age was 75 (70–83) years, and 56.7% of the patients were male.

In a study conducted by Bobula et al, the median length of stay in the cardiology intensive care unit was 22 days.^[10]^ Among the survivors, the length of stay was 21 days, while among those who died in the intensive care unit, it was 35 days. However, our study found that both survivors and deceased patients had a length of stay of 5 days in the cardiology intensive care unit. This shorter length of stay compared to other studies may be due to hospital bed occupancy rates and disruptions in the patient referral chain. Previous research has indicated that prolonged stay in the intensive care unit is linked to higher mortality rates.^[13]^

In our study, the APACHE II score was crucial for determining mortality. The APACHE II score was significantly higher in the deceased group. In our study, high PCT levels were also statistically significant in the dying group. Similarly, Yahav et al reported leukocytosis and PCT levels as essential markers of bacterial infections in elderly patients.^[14]^ In a survey conducted by Xia et al in China, PCT and CRP levels were significantly higher in the positive blood culture group than in the negative blood culture group.^[15]^ In line with the findings of the present study.

Albumin and creatinine levels were also significantly associated with mortality in elderly patients who were followed up in the cardiology intensive care unit.^[16]^ In our study, albumin levels were high in the surviving group and creatinine levels were high in the deceased group. These findings indicate that laboratory values should be considered in the clinical management of elderly patients followed up in the cardiology intensive care unit.

In a study of the causative agents of community-acquired bloodstream infections in elderly patients, the most frequently isolated species were *K pneumoniae* (5.8%), *E faecalis* (4.6%), *Streptococcus pneumoniae* (*S pneumoniae*) (3.7%), and *P aeruginosa* (3.1%).^[17]^ In another study, the most common pathogens among the 264,901 BSI isolates were *S aureus* and *E coli*. *K pneumoniae*, *P aeruginosa*, and *E faecalis* came next. It was found that the number of *E coli* isolates increased, while the number of *S aureus* isolates decreased, and this change was paralleled by an increase in the proportion of GNB among the top 10 pathogens causing BSI.^[18]^ The proportion of *S. pneumoniae* isolates decreased by half.

In our study, the most commonly isolated pathogens in the deceased were *S hominis* (15.6%), *S epidermidis* (14.8%) and *S aureus* (10.9%). The most common gram-negative cocci were *E coli* (9.6%) and *K pneumoniae* (9.6%). The most common gram-positive cocci isolated in surviving patients were *S hominis* (23.5%), *S epidermidis* (24.3%) and *S aureus* (13.4%). The most common gram-negative cocci were *E coli* (5.4%), *K pneumoniae* (5.8%) and *P aeruginosa* (2.5%). These results suggest that the blood cultures grown in the first 48 hours in our cardiology intensive care unit may contain endocarditis agents due to the presence of underlying heart diseases as well as being community-acquired. Although gram-positive cocci were dominant in both groups, gram-negative cocci were seen almost twice as often in the deceased.

BSI is a severe condition that results in death within 1 month of diagnosis in approximately 1-quarter of older people. A study by Hernández et al in the elderly reported a 1-month mortality rate of 21.4% in patients with bacteremia.^[19]^ A study by Nielsen et al in 2015 reported a mortality rate of 45%.^[20]^ Another study found that in-hospital mortality of patients with sepsis in the ICU was 47.3%, compared with 43.6% in other ICUs.^[21]^ In our study, the mortality rate was 48.2%. This rate suggests that patients in cardiology ICU are at a higher risk than those in other ICUs.

These data emphasize the need for caution in managing and prognosis of infection in elderly patients admitted to cardiology intensive care units. In particular, the higher prevalence of gram-negative bacteria in deceased patients necessitates optimization of infection control measures and treatment strategies to target these microorganisms. Furthermore, considering that a prolonged stay in intensive care is associated with increased mortality, it is essential to carefully monitor the length of stay of patients and refer them to appropriate services as soon as possible. In this context, using predictive tools such as the APACHE II score may help accurately assess the mortality risk in elderly patients and determine appropriate treatment approaches.

## 6. Conclusion

With the increase in the elderly population, infection management in elderly patients hospitalized in cardiology intensive care units has become more critical. Our study determined that the APACHE II score is essential for predicting mortality, and that PCT, albumin, and creatinine levels are also associated with mortality. These findings indicate that these parameters should be considered during the early diagnosis and treatment of infections. The fact that gram-negative bacteria are more common in deceased patients necessitates the optimization of infection control measures to target these microorganisms. In conclusion, a multidisciplinary approach should be adopted in the infection management of elderly patients, and risk factors should be carefully evaluated.

## Author contributions

**Conceptualization:** Sibel Doğan Kaya, Yeşim Uygun Kizmaz, Fatih Yiğit.

**Data curation:** Sibel Doğan Kaya, Yeşim Uygun Kizmaz, Fatih Yiğit.

**Formal analysis:** Sibel Doğan Kaya, Yeşim Uygun Kizmaz, Fatih Yiğit.

**Funding acquisition:** Sibel Doğan Kaya, Yeşim Uygun Kizmaz, Fatih Yiğit.

**Investigation:** Sibel Doğan Kaya, Yeşim Uygun Kizmaz, Fatih Yiğit.

**Methodology:** Sibel Doğan Kaya, Yeşim Uygun Kizmaz, Fatih Yiğit.

**Project administration:** Sibel Doğan Kaya, Yeşim Uygun Kizmaz, Fatih Yiğit.

**Resources:** Sibel Doğan Kaya, Yeşim Uygun Kizmaz, Fatih Yiğit.

**Software:** Sibel Doğan Kaya, Yeşim Uygun Kizmaz, Fatih Yiğit.

**Supervision:** Sibel Doğan Kaya, Yeşim Uygun Kizmaz, Fatih Yiğit.

**Validation:** Sibel Doğan Kaya, Yeşim Uygun Kizmaz, Fatih Yiğit.

**Visualization:** Sibel Doğan Kaya, Yeşim Uygun Kizmaz, Fatih Yiğit.

**Writing – original draft:** Sibel Doğan Kaya, Fatih Yiğit.

**Writing – review & editing:** Sibel Doğan Kaya, Fatih Yiğit.
